# Quick Preparation of Moisture-Saturated Carbon Fiber-Reinforced Plastics and Their Accelerated Ageing Tests Using Heat and Moisture

**DOI:** 10.3390/polym8060242

**Published:** 2016-06-22

**Authors:** Masao Kunioka, Tomio Shimada, Hideaki Hagihara, Masahiro Funabashi, Hiroyuki Suda, Hideki Horizono

**Affiliations:** 1 Research Institute for Sustainable Chemistry, National Institute of Advanced Industrial Science and Technology (AIST), Higashi 1-1-1, Tsukuba, Ibaraki 305-8565, Japan; tom-shimada@aist.go.jp (T.S.); h-hagihara@aist.go.jp (H.H.); m.funabashi@aist.go.jp (M.F.); h.suda@aist.go.jp (H.S.); 2 Composite Laboratory, Manufacturing Technology Research Department, Mitsubishi Heavy Industries, Ltd., Takamichi 1, Iwatuka-chou, Nakamura-ku, Nagoya, Aichi 453-8515, Japan; hideki_horizono@mhi.co.jp

**Keywords:** carbon fiber reinforced plastics (CFRP), accelerated ageing test, dynamic mechanical analysis (DMA), electron probe micro-analysis (EPMA), surface and interfacial cutting analysis system (SAICAS)

## Abstract

A quick method involving the control of heat and water vapor pressure for preparing moisture-saturated carbon fiber-reinforced plastics (CFRP, 8 unidirectional prepreg layers, 1.5 mm thickness, epoxy resin) has been developed. The moisture-saturated CFRP sample was obtained at 120 °C and 0.2 MPa water vapor in 72 h by this method using a sterilizer (autoclave). The bending strength and viscoelastic properties measured by a dynamic mechanical analysis (DMA) remained unchanged during repetitive saturation and drying steps. No degradation and molecular structural change occurred. Furthermore an accelerated ageing test with two ageing factors, *i.e.*, heat and moisture was developed and performed at 140–160 °C and 0.36–0.62 MPa water vapor pressure by using a sealed pressure-proof stainless steel vessel (autoclave). The bending strength of the sample decreased from 1107 to 319 MPa at 160 °C and 0.63 MPa water vapor pressure in 9 days. Degraded samples were analyzed by DMA. The degree of degradation for samples was analyzed by DMA. CFRP and degraded CFRP samples were analyzed by using a surface and interfacial cutting analysis system (SAICAS) and an electron probe micro-analyzer (EPMA) equipped in a scanning electron microscope.

## 1. Introduction

Many kinds of carbon fiber-reinforced plastics (CFRP) are used for fabrication of airplanes and in construction due to their low weight and high strength. For example, in the Airbus A380, many CFRP parts are used for reducing weight [1] and CFRP is also used in the manufacture of 50% of Boeing B787 parts [2]. Therefore, in the future more CFRP parts will be used in airplane manufacture. The vertical stabilizer part of Mitsubishi Regional Jet (MRJ) is also produced from CFRP [3]. Furthermore, currently CFRP parts are already being used in high-class commercial automobiles [4]. Toray, a carbon fiber (CF) production company has announced that the market size of CF is expected to double by 2020 [5].

CFRP parts are produced by heating for hardening a thermoset resin such as epoxide with CF prepregs, so a long hardening time (long cycle time) is required. To reduce the process cost by shortening the cycle time, thermoplastics such as polyamide instead of epoxide are used, resulting in carbon fiber reinforced thermoplastics (CFRTP) which can be prepared by the injection molding process [6].

**Figure 1:**
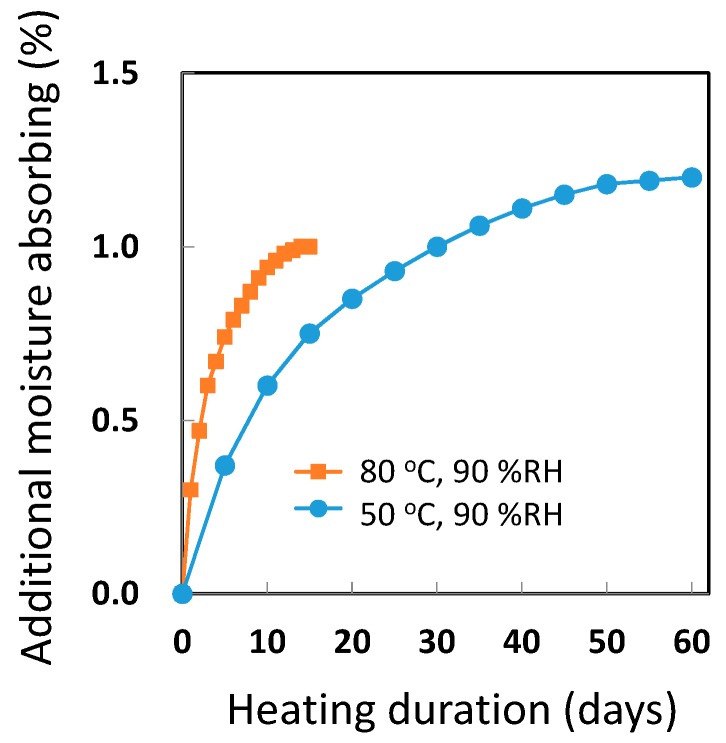
Moisture absorption by carbon fiber-reinforced plastics (CFRP) sample (1.5 mm thickness) at 50 °C and 90 percent relative humidity (% RH) and at 80 °C and 90% RH. Additional moisture absorbing is indicated as percentage to the total mass of non-dry CFRP samples.

For evaluating the mechanical properties of a CFRP part, the properties of moisture-saturated CFRP need to be studied via simulated actual operating conditions. CFRP parts at time of production do not absorb moisture. However, the resin in the CFRP does absorb atmospheric moisture. The amount absorbed by epoxy resins is generally around 5% moisture of their mass. The properties of CFRP change depending on the moisture content. For example, the bending strength of CFRP used in this study was decreased to 90% at moisture-saturated conditions. The moisture-saturated conditions are realized as per ASTM D 5229 [7]. As shown in Figure 1, long absorption durations such as 0.5–2 months are necessary. The equilibrium saturation point is considered to be one at which the difference between the moisture contents (%) at two consecutive measurement points is below 0.02%. For developing and testing effective CFRP, a process to significantly accelerate moisture saturation is necessary.

Lifetime prediction experiments for CFRP based on Arrhenius [8,9,10,11] and non-Arrhenius plots [12,13] are carried out long term to investigate durability. In addition, CFRP samples become dry if the selected measurement temperature is over 100 °C for the highly accelerated ageing test; therefore the contribution of moisture to the degradation is eliminated. The extent of degradation of CFRP under 85 °C and 85% RH is quite small for long evaluation durations e.g., 1 or 2 years. For developing a more durable CFRP, accelerated ageing tests at a higher acceleration and at simulated real operating conditions need to be performed. These tests involve two stresses, e.g., heat and moisture [14], heat and UV radiation [15] or heat and residual stress [16]. In our laboratory an accelerated ageing test was performed with the stresses heat and Xenon lamp radiation for a poly(ethylene terephthalate) (PET) film [17] and the results were analyzed by the Eyring model for lifetime prediction.

CFRP parts are durable but the CFRP underwent hydro-degradation and oxo-degradation near room temperature at a very slow rate. CF is stable in CFRP during use at the room temperature but epoxy resin is degraded by water [18,19] or oxygen [20]. The molecular structure of degraded PET and epoxide were analyzed by performing positron annihilation spectrometry (PALS) [21,22] and dynamic mechanical analysis (DMA) [18,19]. DMA is useful to analyze the molecular motion and glass transition temperature in the amorphous phase [23]. Changing the interface between the CF and resin during degradation significantly affects CFRP degradation. It is therefore very important to clarify the mechanism and position of initiation of degradation in CFRP by performing the accelerated ageing test.

In this study, the quick method for preparing moisture-saturated CFRP (8 layers of unidirectional CF, 1.5 mm thickness, epoxy resin) samples at 120 °C and 0.2 MPa water vapor in 72 h by using a sterilizer (autoclave) has been developed. The moisture absorption was analyzed by measuring the moisture content and determining the viscoelastic and mechanical properties of moisture-absorbing CFRP. In addition, an accelerated ageing test by heating and moisture exposure using a sealed pressure-proof stainless vessel (autoclave) were developed for the evaluation of CFRP durability. Furthermore, the degradation of the CFRP surface and material underneath the surface was characterized.

## 2. Materials and Methods

### 2.1. CFRP Sample

**Figure 2:**
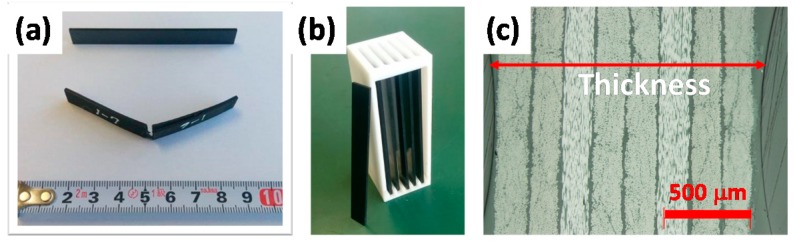
CFRP samples (**a**) CFRP test pieces for bending strength; (**b**) CFRP samples for sealed stemless autoclave; (**c**) cross-section image of CFRP.

CFRP samples, composed of epoxy resin and 8 layers of unidirectional 2-μm-diameter CF oriented at +45, 90, −45, 0, 0, −45, 90 and +45° (fiber content of 72 mass % and thickness: 1.5 mm; see Figure 2) were prepared under 180 °C, 0.7 MPa over 2 h. CFRP samples for bending strength measurement and DMA were cut to 15 mm× 100 mm or 10 mm× 40 mm sizes.

### 2.2. Quick Preparation of Moisture-Saturated CFRP

CFRP samples were moisture saturated in a sterilizer LSX-500 (Inside volume 50 L, TOMY SEIKO CO., LTD., Tokyo, Japan) at 120 °C and 0.2 MPa water vapor pressure for 12–96 h. First, this sterilizer was heated in water to 100 °C and then the air inside was removed by passing steam. Then it was heated again under sealed conditions and 120 °C and 0.2 MPa water vapor pressure was used. The moisture content of CFRP samples was determined by calculating the difference between the wet CFRP mass and dry CFRP mass, measured after drying under a reduced pressure (around 0.1 kPa) at 100 °C for 3 days.

### 2.3. Stability Test

To confirm that the CFRP sample did not undergo degradation or change of in the molecular structure of the epoxy resin during this quick saturation method, the saturation (120 °C, 0.2 MPa water vapor, 72 h) and drying (100 °C, reduced pressure, 72 h) steps were repeated sequentially three times. At the end of each of the three cycles, the moisture content, bending strength and viscoelastic properties were measured.

### 2.4. Accelerated Ageing Test

**Figure 3:**
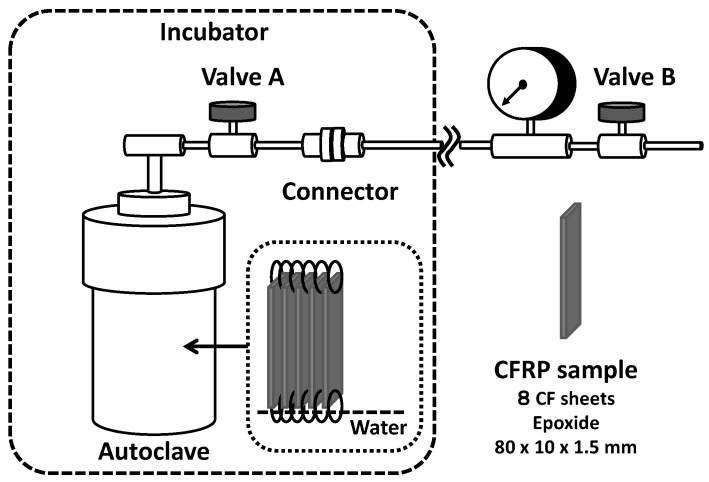
Stainless steel autoclave apparatus for accelerated ageing test for CFRP samples, subjected to moisture exposure and heating.

Accelerated ageing tests for CFRP samples were performed using a sealed pressure-proof vessel (300 mL stainless autoclave, Taiatsu Techno, Tokyo, Japan) with a connecter, valves and a pressure gauge as shown in Figure 3. CFRP samples were placed inside by a fixed Teflon fitting part, not touching each surface of samples, as indicated in Figure 2b, with 20 mL deionized water. When the temperature of water inside reached boiling point (100 °C) at atmospheric pressure after setting autoclave in incubator, valve B was opened for a while to expel the air inside by steam. Then, valve B was closed to maintain the sealed condition and the autoclave was heated to a specific temperature (140, 150, or 160 °C). Valve A was closed after a specific period, the autoclave was removed at the connecter and the autoclave as taken out from the incubator. Then, resultant degraded CFRP samples were taken out after cooling to room temperature.

### 2.5. Mechanical Test

Three-point bend tests of the CFRP samples (15 mm× 100 mm× 1.5 mm) were measured (triplicate) by using the Instron model 3342 (Instron, Norwood, MA, USA) with 6 cm lower span gap and 5 mm/min crosshead speed. The bending strengths were measured for dry CFRP samples after drying at 100 °C under a reduced pressure for 72 h.

### 2.6. Dynamic Mechanical Analysis

Viscoelastic properties of CFRP samples (10 mm× 40 mm× 1.5 mm) were measured by Q800DMA (TA Instrument, New Castle, DE, USA) with 3 °C/min rate of increase in the temperature to 300 °C, 1 Hz frequency and amplitude of 15 μm at the free-end of the cantilever for the bending mode. The sample was clamped at one end and flexed at 27 mm position by single cantilever.

### 2.7. Optical Microscopy

The CFRP sample was roughly cut by using a wet slow thread sawing machine and fixed in acrylic resin to obtain the cross section. After rough grinding with a water-proof sand paper, the surface was polished by a grinder machine MA-150 (Musashino Denshi, Tokyo, Japan) with diamond powder (around 1 μm). Optical microscopy images were obtained by using the BX-60 microscope (Olympus, Tokyo, Japan).

**Figure 4:**
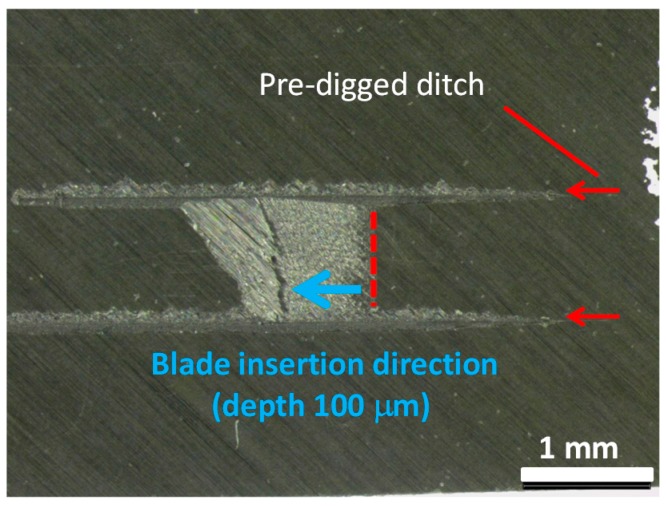
Cutting diagonally from the surface of CFRP sample by using the surface and interfacial cutting analysis system (SAICAS).

The surface and interfacial cutting analysis system (SAICAS) method involves destructive inspection, but since the destroyed area is very small, it can be said to be a non-destructive inspection method. Slant-cut sections from the original CFRP surface were cut by the SAICAS with a diamond blade at horizontal speed of 1.0 μm/min and vertical speed of 0.1 μm/min. Before slant cutting, two parallel ditches (at a distance of 1 mm) were dug to avoid the friction between the side wall and blade as indicated in Figure 4. These ditches prevent friction between the blade and sidewall of the CFRP sample. The diamond blade was inserted from the surface and cut at an angle into the CFRP sample. The cut rate ratio of horizontal and vertical is 10: *i.e.*, if the blade moved 1 mm horizontally, the depth position of the blade was 100 μm. Apparent shear strengths were calculated from the slope of the force curves between 0 and 10 μm depth [24].

### 2.8. Electron Probe Micro-Analysis

**Figure 5:**
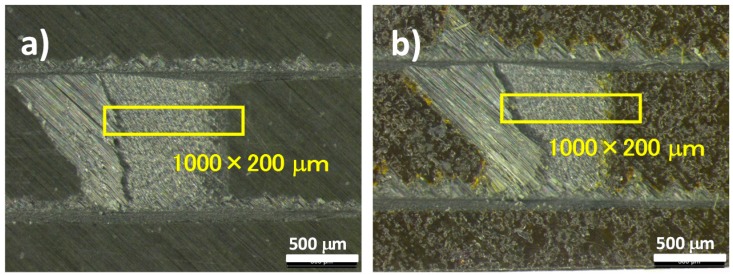
Measuring positions of (**a**) CFRP and (**b**) degraded CFRP samples (160 °C in 14 days with moisture) for electron probe micro-analysis (EPMA).

Slant cutting planes of the CFRP samples were analyzed by using a scanning electron microscope with an electron probe micro-analyzer using JX-8200 (JEOL, Tokyo, Japan). Planes as indicated in Figure 5 were analyzed by EPMA after vapor depositions of thin carbon, platinum and palladium for the existences of sulfur, carbon and oxygen atoms.

## 3. Results and Discussion

### 3.1. Quick Preparation of Moisture-Saturated CFRP

**Figure 6:**
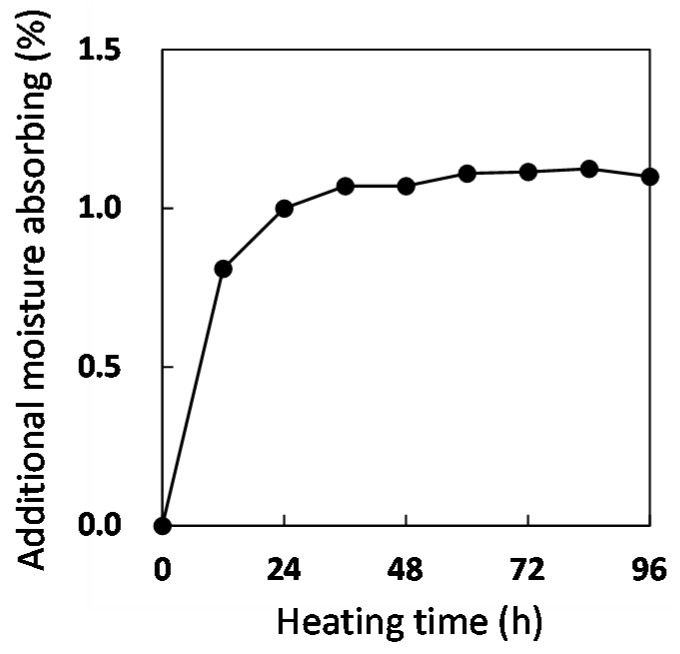
Additional moisture absorbed by non-dry CFRP samples at 120 °C.

Figure 6 shows that the moisture content of the CFRP sample depends on the heating time in the autoclave. Samples were taken at different continuous heating times with different samples used for wet mass measurements in order to avoid the heat cycles between 120 °C and room temperature at mass measuring. It was confirmed that the moisture saturation (1.14%) was reached after 60 h. This saturation rate was faster than that as indicated in Figure 1 because the diffusion rate of water molecules in epoxy resin at 120 °C was higher than that at 50 and 80 °C and the moisture amount (0.2 MPa at 120 °C) of the CFRP surface was much higher than that at 80 °C (0.047 MPa × 90% RH). However, if degradation or structure changes occur in the CFRP, this saturation method cannot be deemed appropriate in simulated conditions as in the actual use environment for CFRP materials.

### 3.2. Stability Test

**Figure 7:**
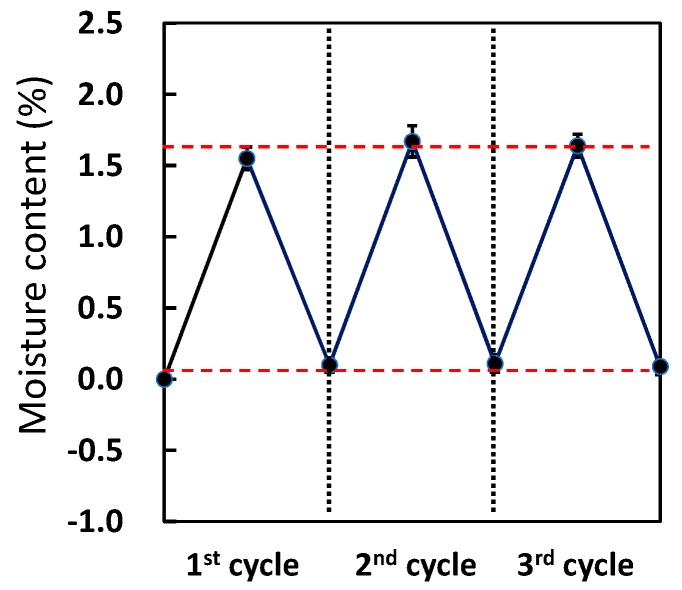
Moisture content of dry CFRP samples during repetitive moisture absorption at 120 °C for 72 h and drying at 100 °C for 72 h under a reduced pressure.

**Figure 8:**
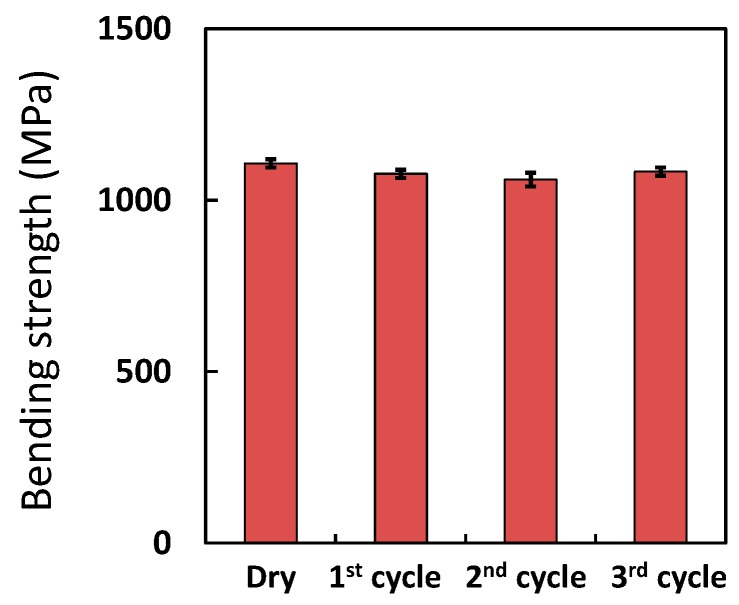
Bending strength of CFRP samples during repetitive moisture absorption at 120 °C and drying.

As shown in Figure 7, the moisture content of the saturated and dry samples after the first cycle were only slightly lower—by 0.1%—than those after the second and third cycles. This confirmed that moisture absorption by CFRP did not change during the three cycles of saturation and drying. The bending strengths of dry CFRP samples after each cycle were almost identical, as indicated in Figure 8. Thus, degradation of CFRP was not detected after the three cycles.

### 3.3. Viscoelastic Properties of Moisture-Saturated CFRP by DMA

**Figure 9:**
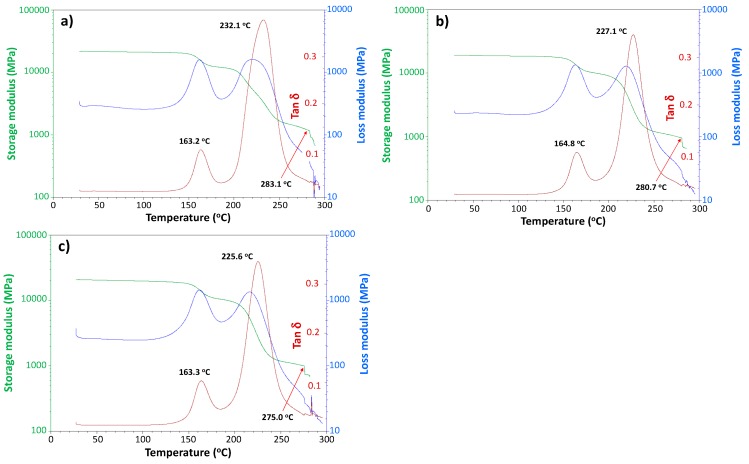
Dynamic mechanical analysis (DMA) charts for dry CFRP sample; (**a**) before moisture saturation and after; (**b**) the first (120 °C, 72 h); (**c**) third moisture saturation cycles. The red line indicates the position that noises are appeared due to the delamination of CF layers.

To confirm that the original untreated CFRP specimen (Figure 9a) and the dry CFRP sample, after the first (Figure 9b) and third (Figure 9c) moisture saturation cycles have the same viscoelasticity, these samples were measured by DMA. The glass transition temperature, *T*_g_ values of the epoxy resin of the CFRP samples (determined by tanδ peak) did not change significantly: 163.2, 164.8, and 163.3 °C, respectively. In addition, other unassigned tanδ peaks remained unchanged as well: 232.1, 227.1 and 225.6 °C. During temperature increase in DMA, noise originating from slight peeling off at layer interfaces due to CFRP delamination appeared at 283.1, 180.7 and 275.0 °C.

Thus, this method does not cause the degradation and change in the molecular structure because of the negligible contribution by hydro-degradation of epoxy resin.

### 3.4. Accelerated Ageing Test

**Figure 10:**
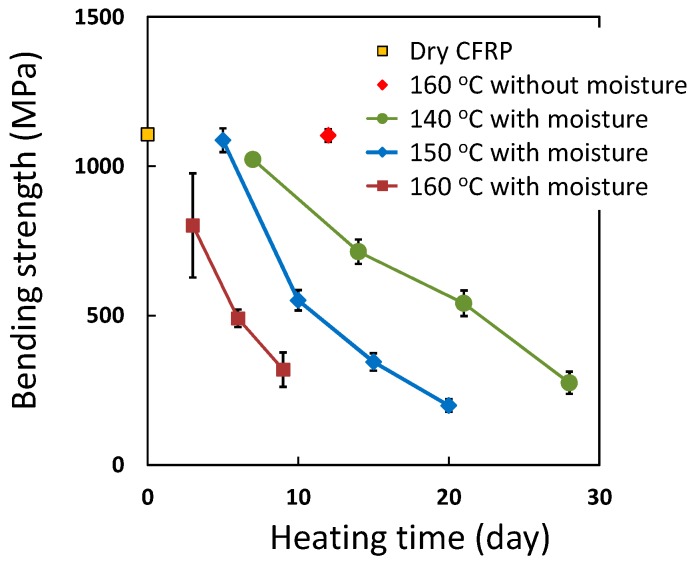
Bending strength of degraded CFRP samples during heating with moisture at 140, 150 and 160 °C and bending strength at 160 °C without moisture for 12 days.

**Figure 11:**
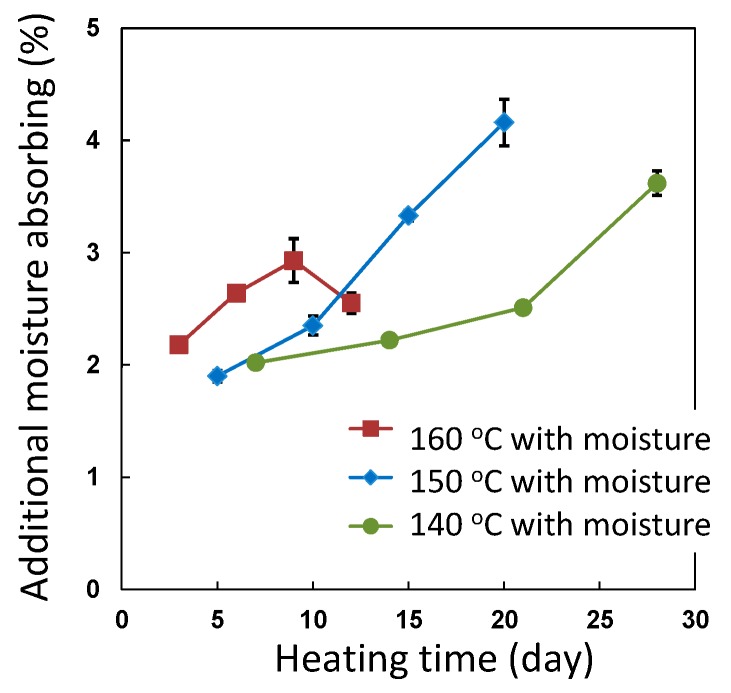
The additional moisture absorbing of degraded CFRP samples during heating with moisture at 140, 150 and 160 °C and moisture content at 120 °C with moisture (quick moisture saturated condition).

Saturated water vapor pressure inside the vessel becomes 0.36 MPa at 140 °C, 0.48 MPa at 150 °C and 0.62 MPa at 160 °C. The bending strength values of the degraded CFRP sample at 140–160 °C with moisture and at 160 °C without moisture over 12 days are plotted in Figure 10. To avoid the effects of decreasing strength due to moisture absorption, these degraded samples were dried at 100 °C under reduced pressure for three days and the bending strength was measured. In the case of the CFRP sample at 160 °C without moisture for 12 days, the bending strength did not decrease. In the case of heating with moisture at 140–160 °C, the bending strength decreased with heating time. CFRP samples were degraded at 140–160 °C with moisture and the rate of degradation was accelerated by increasing the heating temperature. The bending strength of CFRP was decreased from 1107 to 319 MPa at 160 °C and 0.62 MPa for nine days. The additional moisture absorption of the degraded CFRP samples are indicated in Figure 11. The moisture content increased with the increase in the degree of degradation. However, in the case of exposure to moisture at 160 °C, the moisture content after 12 days was lower than that after 9 days. The bending strength of this sample after 12 days could not be measured owing to delamination, due to heavy degradation. In addition, the water inside the autoclave contained precipitates. These observations indicate that a part of this CFRP sample degraded and dissolved in the water, so the mass of this CFRP sample decreased and the moisture content may have decreased as a result.

**Figure 12:**
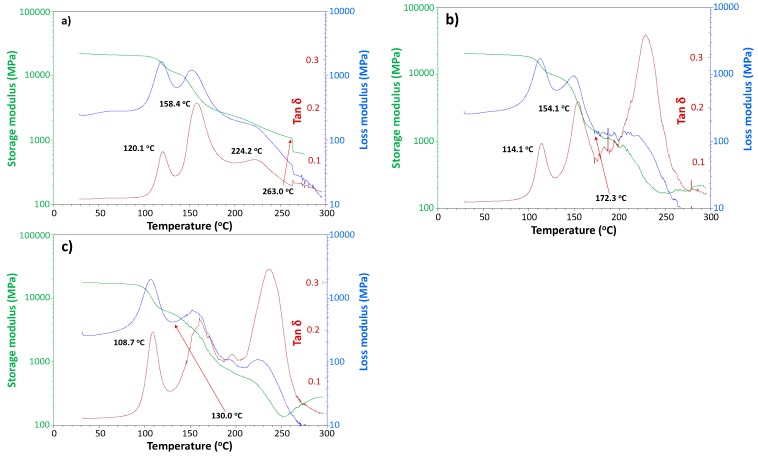
DMA charts of moisture-absorbed CFRP samples at 120 °C in 4 days (**a**), 140 °C in 15 days (**b**) and 160 °C in 7 days (**c**) with moisture.

To clarify the molecular motion properties especially in the amorphous phase, the degraded CFRP samples were subjected to DMA (Figure 12). The bending strengths of these samples were 1107 MPa (original specimen), 714 MPa (specimen subjected to 140 °C with moisture for 14 days), and 491 MPa (specimen subjected to 160 °C with moisture for 7 days). These DMA charts shown in Figure 12, were obtained for moisture absorbing samples after degradation without drying and these are different from the DMA charts of dry CFRP samples shown in Figure 9. Via DMA, the properties of CFRP with absorbed moisture can be obtained during heating conditions with drying in the DMA apparatus. The temperature when noise commenced, due to the delamination of CF layers during heating process in DMA, was observed. In the case of the original CFRP sample, this temperature was 263.0 °C and it decreased to 172.3 or 130.0 °C with the progress in the degradation.

This delamination starting temperature during heating in the DMA apparatus can be used as the indicator of degradability, or heat resistance limit for CFRP samples. The tanδ peak around 160 °C, which is the glass transition temperature of epoxy resin, decreased in height and became indistinct with the progress in degradation due to increased molecular motion with shorter molecules produced by hydro-degradation. Furthermore, the tanδ peak around 120 °C shifted to a lower temperature with the progress in the degradation.

### 3.5. Analysis for Degradation by Optical Microscopy and Scanning Electron Microscopy (SEM) with Electron Probe Micro-Analyzer

**Figure 13:**
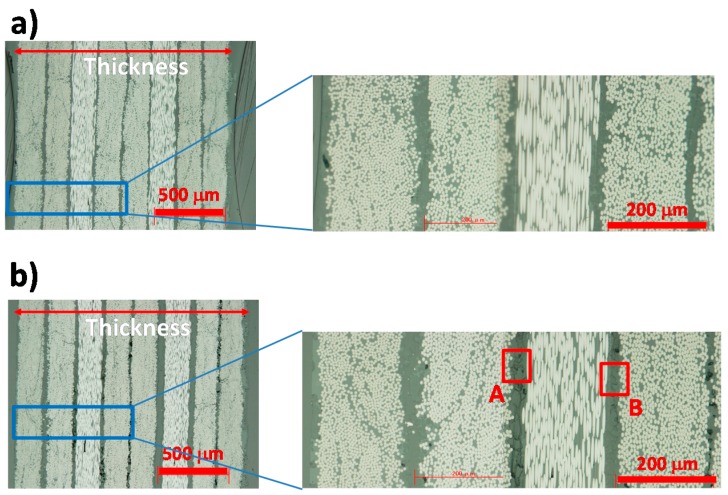
Optical microscopy images for (**a**) CFRP and (**b**) degraded CFRP (160 °C and 0.62 MPa (H_2_O vapor) in 14 days) samples. Expansion images at A and B areas are shown in Figure 14.

**Figure 14:**
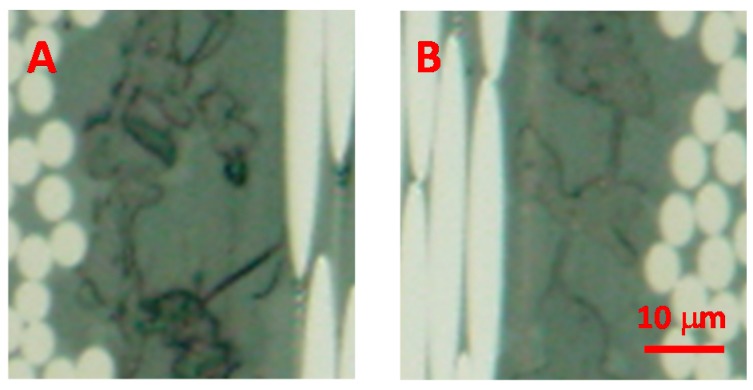
Magnified images of degraded CFRP sample (positions **A** and **B** in Figure 13).

Figure 13 shows optical microscope images of the cross-section of the original and degraded CFRP (160 °C with moisture for 14 days). As indicated in the magnified images in Figure 14, many holes and cracks existed at the interfaces between the 8 CF layers, not only near the surface but also at the center of the sample. The thickness of CFRP sample increased from 1.50 to 1.62 mm after degradation as shown.

**Figure 15:**
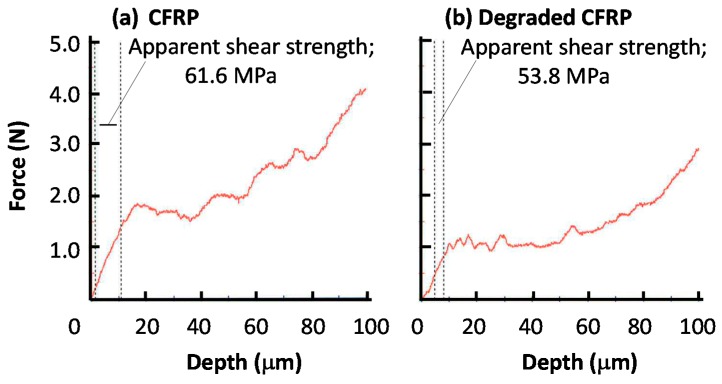
Force during slant cutting by using the surface and interfacial cutting analysis system (SAICAS) for the original CFRP (**a**) and degraded CFRP (**b**) samples.

Figure 14 shows the SAICAS measurement point indicated by red dotted line. SAICAS can measure the cutting force of a blade for a CFRP sample, as indicated in Figure 15. The apparent shear strength can be calculated from the indicated area of the plot of depth *versus* force, as indicated in equation (1) [24].

Apparent shear strength = *F*_h_/2*wd* cot ϕ(MPa) (1)

where *F*_h_, *w*, *d*, and ϕ are changing amount of vertical force (N), width of blade (mm), depth of insertion (mm) and shear angle of blade (45°) respectively. The apparent shear strength decreased from 61.6 to 53.8 MPa at the 0–10 μm depth because of degradation of the CFRP sample. In this depth area only the epoxy resin exists, so these apparent shear strengths are those of the epoxy resin. The bending strength decreased to one sixth that of the original CFRP specimen. However, the strength of the epoxy resin decreased by only 12%. The decrease in bulk strength (more than 70%, 1107 MPa to 300 MPa at nine days and 160 °C as indicated in Figure 10), such as bending strength was much higher than in micro strength such as resin shear strength (12%) or interfacial adhesion strength, between CF and resin (44%, 1.8 N to 1.0 N, force in 20–60 μm depth by SAICAS as indicated in Figure 15). A small decrease in the micro strength results in a significant decrease in the bulk strength; in other words, microcracks significantly affect the bulk strength.

In addition, the cutting force at 20–80 μm depth was decreased from 1.8 to 1.0 N by degradation of the CFRP sample. This depth region corresponds to the CF layer. The strength of CF may not be affected by this condition (160 °C and 0.62 MPa, H_2_O vapor, after 14 days). However, the strength of resin and interface adhesion between the resin and CF may decrease. Thus, it was confirmed that by using the SAICAS, the degradability can be estimated from a very small part of the CFRP sample.

Figure 15 shows the measuring positions for SEM (with EPMA) images of the slant-cut surfaces of original and degraded CFRP samples: the surface was damaged by degradation and there were many crater holes, Figure 15b. The thickness of the epoxy resin layer between the surface and the first CF layer decreased from 10 to 5 μm.

**Figure 16:**
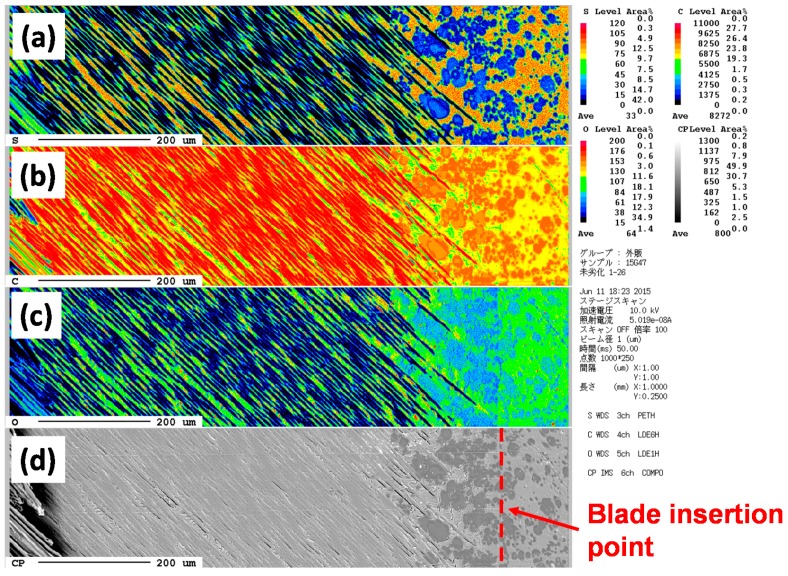
EPMA mapping of CFRP sample for sulfur (**a**), carbon (**b**) and oxygen atoms (**c**). Image (**d**) shows the SEM image with the marked measurement position.

**Figure 17:**
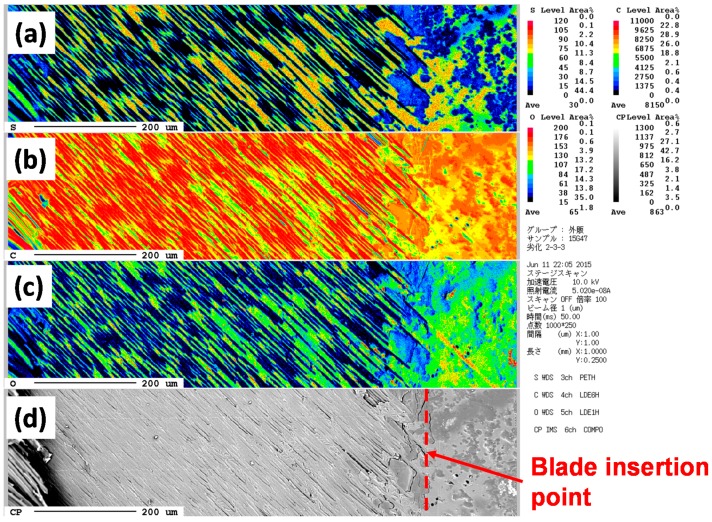
EPMA mapping of degraded CFRP sample (160 °C in 14 days with moisture) for sulfur (**a**), carbon (**b**) and oxygen atoms (**c**). Image (**d**) shows the SEM image with the marked measurement position.

Figure 16 and Figure 17 show the sulfur, carbon and oxygen mapping and the SEM images of the original and degraded CFRP (160 °C with moisture for 14 days). From the differences between the maps of the original and degraded CFRP samples, the amount of sulfur atoms were found to have decreased at the surface and near the surface, as indicated by the color change from orange, right-side area of Figure 16a to green and blue, Figure 17a. This component with sulfur existed as a phase separation component that may be a modifier such as poly(ether sulfone). This component is hydrophilic but can absorb small amounts of water with a resultant change in properties. This component was eliminated from the surface via degradation of the CFRP sample. The number of carbon atoms at the surface slightly increased, as indicated by the color change from yellow to orange, Figure 16b and Figure 17b. This may be due to the appearance of epoxy resin by removal of the sulfur-containing component. The amount of oxygen atoms at the surface increased, as indicated by the color change from green to yellow, Figure 16c and Figure 17c, because the component with sulfur was removed due to the same reason as that for carbon (mentioned above) and the surface was damaged by oxidation or hydro-degradation. Furthermore, the number of oxygen-containing functional groups such as carboxyl or hydroxyl groups may have been increased. However, in the CF layers, the ratio of these atoms did not change.

In the near future, the changing in the narrow area in these maps will be analyzed in detail, so as to more clearly identify and investigate the molecular changes.

## 4. Conclusion

By heating the CFRP sample at 120 °C with water vapor (0.2 MPa) for 72 h, a moisture-saturated sample without any degradation or change in molecular structure was obtained. This newly developed, quick method for moisture-saturated CFRP preparation, which to date required a long duration under simulated actual environmental conditions, can help shorten the R&D time. In addition, in the new accelerated ageing test method, heating the specimen with moisture at 140–160 °C can degrade CFRP in a shorter duration of around 3–30 days as indicated in Figure 10. Over this period, the bending strength decreased to one sixth of the original value. The degradation and degradability of the CFRP samples could be investigated by using DMA and SAICAS. Degradation was also studied using SEM with EPMA. It was confirmed that the sulfur-containing component was removed during the accelerated ageing test. At this moment, the mechanisms of hydro-degradation, the acceleration degree and the change in the molecular structure of CFRP samples is not completely clear and so in the near future, the degraded CFRP samples will be analyzed in detail. Thus, these methods are useful for R&D to accelerate the development of more stable CFRP materials for hydro-degradation.
